# Supplementary figures and images for: Fine-grained simulations of the microenvironment of vascularized tumours
Source: Sci Rep. 2019 Aug 12;9:11698. doi: 10.1038/s41598-019-48252-8 (PMC6690935; doi:10.1038/s41598-019-48252-8)

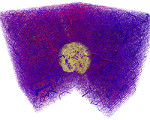

Supplement: Supplementary file 3 — Tumorcode source files [file 41598_2019_48252_MOESM3_ESM.zip › tumorcode-master/doc/tumor.jpg]
